# Key questions: research priorities for student mental health

**DOI:** 10.1192/bjo.2022.61

**Published:** 2022-05-10

**Authors:** Katie Sampson, Michael Priestley, Alyson L. Dodd, Emma Broglia, Til Wykes, Dan Robotham, Katie Tyrrell, Marta Ortega Vega, Nicola C. Byrom

**Affiliations:** Institute of Psychiatry, Psychology and Neurosciences, King's College London, UK; School of Education, Durham University, UK; Department of Psychology, Northumbria University, UK; School of Psychology, University of Sheffield, UK; Institute of Psychiatry, Psychology and Neurosciences, King's College London, UK; The McPin Foundation, UK; Research Directorate, University of Suffolk, UK; Maudsley Learning, South London & Maudsley NHS Foundation Trust, UK; Department of Psychology, Institute of Psychiatry, Psychology and Neurosciences, King's College London, UK

**Keywords:** Student mental health, priority setting, qualitative research, prevention

## Abstract

**Background:**

The high prevalence of mental distress among university students is gaining academic, policy and public attention. As the volume of research into student mental health increases, it is important to involve students to ensure that the evidence produced can translate into meaningful improvements.

**Aims:**

For the first time, we consult UK students about their research priorities on student mental health.

**Method:**

This priority setting exercise involved current UK university students who were asked to submit three research questions relating to student mental health. Responses were aggregated into themes through content analysis and considered in the context of existing research. Students were involved throughout the project, including inception, design, recruitment, analysis and dissemination.

**Results:**

UK university students (*N* = 385) submitted 991 questions, categorised into seven themes: epidemiology, causes and risk factors, academic factors and work–life balance, sense of belonging, intervention and services, mental health literacy and consequences. Across themes, respondents highlighted the importance of understanding the experience of minority groups.

**Conclusions:**

Students are interested in understanding the causes and consequences of poor mental health at university, across academic and social domains. They would like to improve staff and students’ knowledge about mental health, and have access to evidence-based support. Future research should take a broad lens to evaluate interventions; considering how services are designed and delivered, and investigating institutional and behavioural barriers to accessibility, including how this varies across different groups within the student population.

In the context of increasing prevalence of youth and young adult mental health problems,^1,2^ including university students,^3^ concern about mental health in the university setting is mounting and gaining media and public attention.^4^ Increasing demand for services on campus has been observed internationally.^2,3^ However, current approaches lack a solid evidence base,^5,6^ and students have voiced concerns that existing services do not meet their needs.^7^ In the UK, representatives of university leadership and students are urging the sector to adopt a whole-institution approach.^8,9^ However, questions about how to achieve this remain unanswered. Eliciting student perspectives and experiences has been highlighted as an enabling strategy for the sector to develop effective and targeted initiatives attuned to diverse student needs and situated within a whole-university approach.^9^ As research efforts mount,^10^ it is important to involve students to ensure that work in this field translates into meaningful improvements attuned to students’ lived experiences.^11^ This project set out to consult students in the UK on their priorities for future research into student mental health. Our aim is to ensure that the student voice is influential in shaping the direction of future research.

## Method

### Lived experience involvement

The project was initiated through the UK Research and Innovation funded Student Mental Health Research Network (SMaRteN), with a steering group developed from the SMaRteN leadership team. The group recruited diverse stakeholders, including students (both with and without lived experience of mental health difficulties at university), clinical psychologists, tutors and academic researchers. Co-creation was central to this project. This is distinguished from student consultation and participation, by the active involvement of students as equal stakeholders,^12^ reciprocally sharing knowledge and networks as part of a strengths and asset-based approach.^13^ Students were operating in a ‘peer researcher’ context, and worked with academic researchers to design the methodology, recruit a diverse student sample, analyse data and write up the findings. Several student peer researchers are authors on this paper.

### Participants

All procedures contributing to this work comply with the ethical standards of the relevant national and institutional committees on human experimentation and with the Helsinki Declaration of 1975, as revised in 2008. All procedures involving human participants were approved by the university ethics board (approval number LRS-19/20-14288). All participants were provided with information about the study and the opportunity to contact the researchers to ask questions before providing informed consent through an online form.

Our sample included 385 UK university students, who responded to advertisements publicised by SMaRteN and the student mental health charity, Student Minds. Advertisements were circulated through newsletters, Facebook, Twitter and Instagram. We did not provide any monetary incentive for participation. Adverts and the study information sheet reminded participants that the survey would provide an opportunity to help shape the future of research in this area, with SMaRteN funding being allocated to address the top priority questions.

Our sample was primarily under 25 years old (*n* = 285, 74%) and mostly comprised women (*n* = 251, 65%). Our sample included UK domiciled (*n* = 279, 72%) and international (*n* = 106, 28%) students and was 72% White, 15% Asian and 6% Black. A substantial minority of respondents identified as a sexual or romantic minority (*n* = 118, 31%) and/or reported having a disability (*n* = 95, 25%). Students represented all years and levels of study (undergraduate, 66%; taught postgraduate, 17%; postgraduate research, 15%), and represented most subject areas (see Table 1).

**Table 1:** Sample representation across academic areas, compared with UK representation, as reported in Higher Education Statistics Agency data

Academic area	Our sample (%)	UK university students (%)
Medicine and dentistry	9	3
Subjects allied to medicine	9	12
Biological sciences	22	10
Veterinary science	−	<1
Agriculture and related subjects	1	1
Physical sciences	6	4
Mathematical sciences	3	2
Computer science	10	5
Engineering and technology	2	7
Architecture, building and planning	−	2
Total sciences	62	46
Social studies	12	10
Law	3	4
Business and administrative studies	3	15
Mass communications and documentation	5	2
Languages	5	4
Historical and philosophical studies	7	3
Creative arts and design	4	8
Education	4	6
Total non-sciences	38	54

### Procedure

Data collection was carried out via an online survey hosted on Qualtrics (Seattle, WA, USA; see https://www.qualtrics.com/uk/) between October 2019 and February 2020. The survey was designed to be as short and simple as possible to make participation as easy. Respondents submitted up to three questions in response to the prompt: ‘In terms of student mental health, what do you think are the priority issues for researchers to explore?’. There was no word limit for the respondents’ submissions. After submitting questions, respondents were asked to complete demographic details.

### Data analysis

The objective of our analysis was to understand respondents’ recommendations for future research and categorise these to create a shortlist of research priorities. We sought to capture student recommendations without passing judgement regarding the value of the research topic or whether the question had already been addressed.

A team of 26 students were involved in analysing the data, supported by experienced researchers. The student team were recruited through SMaRteN from universities across the UK. Selection focused on bringing together a diverse team. SMaRteN hosted a 2-day workshop, covering expenses to bring students together for training and co-creation activities.

**Fig. 1:**
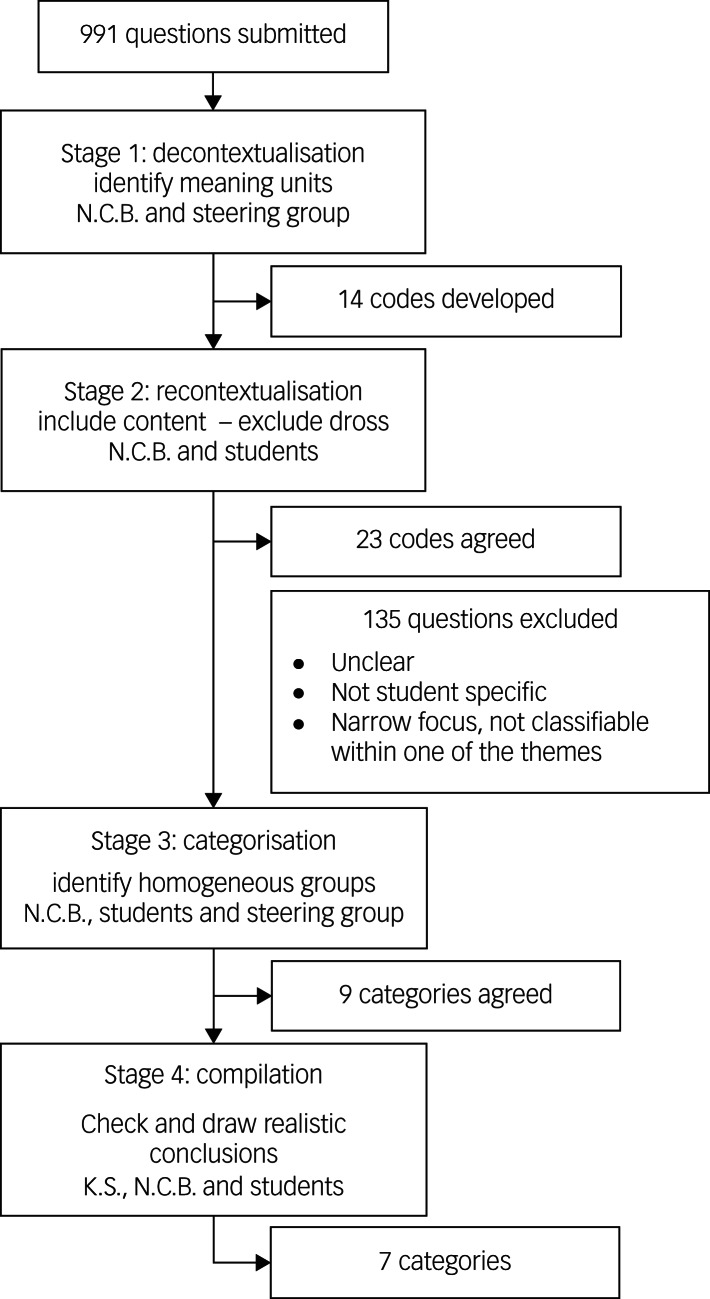
Content analysis process.

To facilitate reflexivity,^14^ we followed an iterative approach, with themes developed and refined through consultation with all members of the team. This improves the reliability of analysis, minimising biases arising from individual researchers’ preconceptions. As respondents were invited to provide single sentence questions, without explaining their rationale, it was important not to overanalyse the data. In this context, content analysis was appropriate. As summarised in Fig. 1, we followed four steps of content analysis, embedding the principles of co-creation in each step.^15^

In stage 1, codes were generated inductively, working with the questions provided rather than bringing in any preconceived ideas of the research questions that might be important. This approach was adopted to ensure we think carefully about the questions students were asking, as opposed to trying to fit their questions into the existing research framework. This process was completed independently by members of the steering group, before in-depth discussion, through which a single list of codes was agreed.^14^

At stage 2, students checked that all aspects of the content had been covered by revisiting the original questions, determining what should be included and excluded, and developing more detailed codes.^15^ For example, although the initial list of codes had included ‘academic pressure’, student analysis here clarified that this should include all questions related academic grades, success, workload, deadline and course-specific challenges.

In stage 3, the lead researcher (N.C.B.) worked iteratively with small groups of students to create categories around the codes, with the goal of reducing the categories without losing the content of units.^16^ Returning to the example of academic pressure, we identified parallels between the questions that had been grouped into this code and questions relating to the university extenuating/mitigating circumstances process. Students agreed it was hard to consider the impact of extenuating circumstances without considering these in the wider context of academic culture and assessment practice. Further, most questions relating to work–life balance focused on managing workload, and hence had clear relationship to the questions grouped under academic pressure. As such, we reduced the number of categories by grouping questions together into the category of ‘academic factors’. This process was continuously appraised to ensure categories were internally homogenous and externally heterogenous.^17^ For instance, although the questions around academic pressure and extenuating circumstances align, questions relating to academics’ appreciation for the pressure students experienced aligned more clearly with other questions about mental health literacy and academics’ understanding of mental health. In the final stage, categories were checked, named and described.

Across the analysis, although we primarily followed a manifest analysis, describing respondents’ questions as they were presented, at times a more latent approach was necessary to interpret questions that were phrased less clearly.^13^ For example, the question ‘What is the effect of workload on students’ mental health?’ can be simply described as asking about student workload, and thus grouped with other questions around workload and academic pressure. In contrast, we received a question reading ‘the amount of work?’. We chose to retain this question and place it within the category of ‘academic factors and work–life balance’. However, here we have assumed the respondent is referring to the work students have to do, rather than the amount of work universities might have to put in to improve student mental health.

## Results

In total, 991 questions were submitted and arranged into seven categories. In Table 2, these categories are set out in descending order of frequency based on the number of questions asked in that category. We discuss each of these categories below.

**Table 2:** Summary of key priorities in context of existing research

Research priority (number of questions asked)	Current evidence	Direction for future research
Intervention and services (*n* = 224)	Short-term embedded counselling at university shows clinical efficacy. Limited evidence suggests non-clinical interventions may be effective	How effective and accessible are university mental health services for a diverse student population? How effective is a whole-university approach to mental health? What is the impact of collaboration between universities and the NHS [National Health Service]? Are non-clinical interventions, e.g. yoga and exercise, beneficial to the student population?
Academic factors and work–life balance (*n* = 121)	Student workload contributes to stress. Changes to university curricula and pedagogy can have beneficial consequences for student well-being	What are the consequences of making curricula and pedagogy changes, which are mindful of student mental health and well-being, across different types of degrees?
Mental health literacy (*n* = 114)	Programmes to improve mental health literacy may be effective in student populations. Academics are under increased pressure to support student mental health, but many find it challenging to understand their role and the best response	What approaches to mental health literacy programmes are most effective and accessible to students? How can academic staff be supported to recognise and help students with mental health difficulties?
Causes and risk factors (*n* = 107)	Multiple individual risk factors have been associated with poor mental health among students	Can we identify relative risk and protective factors associated with mental health difficulties?
Sense of belonging (*n* = 97)	Loneliness, which has negative consequences for mental health, is associated with transition to adulthood and away from the family home	In what way, and for what reasons, do students experience loneliness and how can it be prevented? How are students from minority groups affected by loneliness and structural exclusion?
Epidemiology (*n* = 68)	Prevalence estimates of mental health problems are 20–40%, and have been increasing in recent years	How does prevalence for mental health problems vary between students, considering institution, year of study, academic discipline and minority groups?
Consequences (*n* = 26)	Mental health affects education achievement at university	Do mental health difficulties among students have long-term implications for social and career development?

### Intervention and services

The efficacy of existing services (including counselling, workshops and drop-in services) was raised, including whether these services meet the needs of a diverse student population. Respondents suggested the potential effects of a broad range of specific and sometimes novel interventions, including physical activity, yoga, mindfulness, social activities and events, and sleeping pods on campus. Questions considered cost-efficacy as well as how to increase funding.

Respondents questioned the appropriate balance between preventative work and responsive treatment, and how university support services should be designed to meet needs ranging from well-being through to complex and enduring mental health problems: ‘How can the support for student well-being versus chronic/severe mental illness be differentiated and acknowledged as separate issues?’

Respondents identified a need to clarify where the boundaries of responsibility between the National Health Service (NHS) and university services should lie and how these services should be better integrated, especially with the split between home and term-time addresses: ‘What is the role of universities in treating, preventing, helping with mental health? Where do they fit in with the NHS, charities and family/social structures?’ Questions asked whether there is adequate provision of professional mental health support for students, whether this is suitably accessible and what steps can be taken to improve availability and accessibility.

### Academic factors and work–life balance

Respondents queried how academic pressure, including challenging content, high workload and a pressure to succeed, contribute to mental health problems. This pressure also included how academic success affects self-worth and how to overcome feelings of shame or embarrassment when struggling academically. Pressure was raised in relation to postgraduate students, with a focus on the relationship between mental health, performance and output. Respondents asked what steps can be taken to help those studying at university to cope with pressure: ‘How can students’ resilience and coping be increased so they are best equipped to deal with HE [higher education] study?’

Participants questions indicated that methods of assessment at university may affect mental health and asked whether changes to assessment design could reduce negative effects. Respondents were interested in examinations versus coursework, as well as how deadlines affect stress. A few questions considered the accessibility and efficacy of university extenuating circumstances: ‘Are universities able/willing to make the more flexible adjustments needed for students with long-term mental health conditions to engage?’

University teaching, including module organisation and structure, number of contact hours and online versus in-person teaching, were raised as potentially affecting mental health. Teaching style changes between school and university were also flagged as possibly problematic: ‘I feel like a lot of people are struggling with the first year. How can we make the gap between uni and high school smaller?’ These questions were raised by students across academic disciplines. Healthcare students uniquely also questioned how placements affect mental health.

Respondents asked about the challenge of time management and maintaining balance in their lives. Questions considered how to balance academic work with a social life and part-time job, and postulated whether trying to achieve this places strain on relationships and well-being. Although there were comparatively few questions relating to balance, students involved in the analysis requested that this theme be highlighted because of its relevance and importance.

### Mental health literacy

Questions included whether, and in what ways, a culture of increased awareness, education and conversation would affect student mental health: ‘How is the growing awareness of mental health impacting student's mental health?’ Students were concerned about to identify mental health problems in themselves and their peers, and asked for more knowledge about how to respond to and help someone struggling with mental health problems. The importance of providing support to those who are helping friends with mental health problems was also highlighted. Students wanted knowledge of self-help strategies, and questioned how best to manage and cope with their own mental health problems at university: ‘What steps can students take to minimise their risk of adverse mental health issues?’ This theme also included whether students know what support and advice is available at university, and how they can access it, including how comfortable people feel reaching out for support, the role of stigma and shame, and how to encourage help-seeking behaviour.

Academic staff also play a part in creating a culture around mental health, and so respondents were interested in their mental health literacy and suggested providing resources, training programmes or policy implementation to help staff recognise and support students with mental health problems. Some questions considered whether students feel they are treated as individuals or in a more depersonalised and anonymous manner, and what impact this has on student mental health: ‘Would students suffering with poor mental health be able to work better with more consideration from teachers?’

### Causes and risk factors

Identifying potential risk and protective factors for poor mental health was highlighted: ‘Which students are most at risk of poor mental health/well-being and why? And most likely to have good mental health and why – protective factors?’ Respondents posed questions about underlying reasons, triggers or drivers for problems, with some assuming that university has a negative impact on mental health: ‘What is causing mental illness at university, and is it a systemic problem?’ Specific possible contributing factors included student finances, living arrangements, drug and alcohol use, unhealthy lifestyles and concerns for future career prospects. Questions about living arrangements considered the impact of living away from home, transitioning between home and term-time addresses, communal versus solitary living and how living in halls of residence affects mental health. Respondents queried how a sense of belonging and academic factors, including the challenge of finding a work–life balance, might contribute to mental health problems. These questions have not been included here because there was sufficient interest to create independent categories.

### Sense of belonging

Respondents wanted to know whether all students feel valued, included and appreciated within their university community, and how to improve this: ‘How can students feel more ‘at home’ and comfortable in their universities?’ Loneliness and isolation were raised, particularly the reasons why students are lonely, how this affects mental health and what can be done to reduce it. Respondents questioned how to make meaningful connections, and why students may feel alone despite being surrounded by people. Respondents considered how student social life affects mental health, including the role of societies and sports groups as well as negative experiences such as peer pressure, elitism and bullying.

Questions considered these problems from the perspective of minority or vulnerable groups, with issues surrounding loneliness being raised specifically for international, mature and commuter students. Respondents queried whether the university environment is inclusive for neurodiverse, minority ethnic, LGBTQ+, working class and disabled students, and how a lack of representation may accentuate loneliness. Victimisation and discrimination, including racism and sexual assault, were identified as potentially contributing to mental health problems at university: ‘How safe do you feel on your campus? Specifically, relevant for minoritized groups, i.e. BAME, LGBTQ, non-neurotypical students, etc. and women’.

### Epidemiology

Questions falling into this category considered the prevalence of mental health problems among university students, including identifying the most common conditions, how the incidence of these problems is changing in universities and how prevalence differs between students and non-students. Many questions revealed underlying presumptions that student mental health is declining, and that students are more vulnerable to mental health problems than their peers: ‘Why has the prevalence of mental health problems in university students increased?’ They also questioned when mental health problems develop, whether this is before or after coming to university and how the move to university changes peoples’ experiences.

### Consequences

A small number of questions asked about the consequences of mental health problems at university, and particularly the impact on academic achievement and social life. Respondents asked about drop-out rates in relation to mental health, and consequences for career development. Respondents were interested in the prognosis for those who struggle with mental health problems at university, including rates of recovery.

## Discussion

The aim of this co-creation project was to identify the mental health research priorities of university students and enable the student voice to shape the direction of future research. Our study identified seven key areas for future research. Many themes overlapped, reflecting the interconnectedness of different facets of student life. As summarised in Table 2, we have positioned the students’ priorities in the context of the existing research, which is often small scale and narrowly focused, with limited consideration of racial, ethnic and sexual minorities. The project was undertaken before the COVID-19 pandemic, which has resulted in substantial disruption to students’ lives and rapid changes to university practices, and highlights the long-term challenges facing student mental health. It is important that student priorities are considered as the higher education sector transitions to a post-pandemic world.

### Intervention and services

Although the data available suggest that short-term embedded counselling at university is clinically effective,^18^ evaluation of the efficacy of university mental health services has been minimal.^19^ There has been limited evaluation of interventions and services as part of a whole-university approach,^5,6^ and to our knowledge, no published evaluation of the impact of collaboration between universities and the NHS. Although there has been some consideration of non-clinical interventions such as yoga and exercise, most studies are of poor quality, and it is not possible to rank which interventions work best, where and for whom.^6^ Future studies must take a broader lens to evaluate interventions for students, especially how they are designed, delivered and made accessible, and should employ robust evaluation of service efficacy. In line with student priorities, it is vital that future research considers the efficacy of services for the diverse student population.

### Academic factors and work–life balance

International research indicates that student workload is a major factor contributing to stress and can result in prolonged study times or drop-out.^20,21^ However, despite growing research interest in a ‘whole of curriculum approach’, knowledge about how to support student mental health through curricula and pedagogy is lacking. Preliminary evidence from the USA demonstrated that a multidimensional curricula intervention involving reduction in contact time, a change in grading system, collaborative and practical learning initiatives, and an embedded resilience and mindfulness intervention, resulted in significant decreases in depressive and anxiety symptoms among medical students, with corresponding increases in quality of life, group cohesion, student satisfaction and examination scores.^22^ This suggests that there is a promising way forward that could be adopted in the UK context across different types of degrees, in keeping with the many student questions on this topic.

### Mental health literacy

Mental health literacy is defined as ‘knowledge and belief about mental disorders which aid their recognition, management or prevention’.^23^ Within this, understanding how to look after your own mental health and support peers is fundamental.^24^ Preliminary research has demonstrated potential efficacy and acceptability of peer support programmes^25^ and programmes to improve student mental health literacy among students.^26^ However, further research is needed to evaluate these more thoroughly and compare different approaches. Academics are under increased pressure to support student mental health, but many find it challenging to understand their role and the best response.^27^ Research findings around mental health literacy are varied, with some studies identifying knowledge gaps^28^ and others noting good levels of literacy among students and staff.^29^ Exploring how staff and students can support themselves and others with mental health difficulties is an important priority for future research.

### Causes and risk factors

Research has only focused on whether a specific factor, in isolation, is relevant to mental health. For example, there is strong evidence of relationships between mental health problems and financial stress,^30,31^ drug and alcohol consumption,^32,33^ isolation and loneliness,^34^ and sleep disruption^35,36^ among students, as well as experiences of adverse events before and during university.^37^ Although studies have increasingly explored the link between factors such as accommodation environments,^38,39^ and physical activity^40^ and student mental health, investigating general and comparative risk, protective and causal factors associated with mental health problems among students remains a high priority.

### Sense of belonging

The repeated use of the word ‘loneliness’ within submitted questions was striking. There are strong links between loneliness and mental health problems,^41^ and loneliness is particularly associated with the transition from adolescence to adulthood.^42^ Loneliness appears to be accentuated by the significant upheaval in social networks that occurs when young adults leave the family home.^43^ Research focusing on loneliness and student and postgraduate mental health is developing,^34,44^ but studies to establish how student friendship groups form, how and why students experience loneliness at university and how student loneliness can be prevented should continue, particularly with student input. The COVID-19 pandemic caused further disruption to students’ social networks, with public concern for students missing the university experience.^45,46^ It will be important for research exploring the impact of COVID-19 to recognise that challenges around sense of belonging on the university campus predate the pandemic.

A sense of belonging is unique to the individual. As recognised by the students in our study, it is vital for issue of belonging and loneliness to be investigated among minority groups. Although there is a substantive body of research on attainment gaps for students from minority ethnic backgrounds in UK higher education,^47^ there are evidence gaps related to how structural exclusion affects mental health.^48^

### Epidemiology

Existing evidence suggests 20–40% of university students are likely to meet criteria for mental health problems, and prevalence rates have been increasing over recent years.^2,3^ Analysis of large population data-sets provides conflicting evidence about the relative prevalence of mental health problems between university students and peers not in higher education.^2,49^ With notable exceptions,^50,51^ there has been limited work within the UK identifying how mental health problems might vary across years of study, academic disciplines and universities, although this has been explored extensively within the USA.^21^ Current data around the prevalence of mental health problems for minority student groups also remains limited.

In line with student priorities, future research must provide more precise estimates of the prevalence of student mental health problems, and identify how these vary across the student population. This will have important implications for service planning and provision. Given many students have pre-existing beliefs regarding prevalence and trends of mental health problems at university, clear communication of existing data and future findings is imperative.

### Consequences

In keeping with students’ concerns, research suggests mental health does affect educational achievement at university.^52–54^ However longitudinal studies assessing long-term consequences across a wider breadth of domains, including social life and future career development, are lacking.

### Strengths and limitations

Student involvement in every stage of the study increased the likelihood that the project would be responsive to students’ needs and research priorities. Our sample was broadly representative of the student population, although it overrepresented women, underrepresented undergraduate students and overrepresented students studying sciences, primarily because of a large representation of students studying medicine and dentistry, biological sciences (including psychology) and computer science (see Table 1). Given the widespread underrepresentation of men in research into student mental health, it will remain important for future research to develop specific strategies to consult and engage male students in research design. As a self-selective sample, it is important to recognise that the voices of students who care passionately about student mental health are likely to have been overrepresented in this project.

In conclusion, this project identifies seven key priorities for future research into student mental health from the perspective of UK university students. Students’ questions are mostly unmet in the existing literature, with less research into the mental health of racial, ethnic and sexual minority student communities. Research is needed in each of these seven areas, and Table 2 highlights key questions to be answered. However, three areas stand out as particularly important. ‘Interventions and services’ was the largest category of questions. This is also an area where there are research gaps. We do not need more research evaluating whether one-to-one clinical interventions are effective. Rather, research needs to assess the whole-university approach, understand the range of needs across a diverse student population and consider the broad student experience of services, from initial help-seeking through identifying appropriate support, triaging, waiting lists and using the service. In contrast to the attention students have given to academic factors, the research in this area is sparce. We need robust, large-scale evaluations of the impact of curricula and pedagogy on student mental health. Finally, there is a stark gap between student interest and research exploring sense of belonging. Future research must address the university social experience, to enhance our understanding of how this relates to student mental health and how it might be leveraged to improve mental health.

Our results have important implications for future funding to ensure research produces knowledge that is useful, relevant and meaningful to diverse student populations, as well as ensuring that knowledge can be translated into positive and practical changes within the higher education sector.

## Data availability

The fully anonymised data that support the findings of this study are available online from figshare at https://doi.org/10.6084/m9.figshare.15124908.

## References

[1] McManus S. General Population Surveys: Comparing Student and Non-Student Mental Health. Kings College London, 2019 (https://kclpure.kcl.ac.uk/portal/en/publications/general-population-surveys(5f7c10f4-b901-441d-878f-55a9826e725e).html).

[2] Tabor E, Patalay P, Bann D. Mental health in higher education students and non-students: evidence from a nationally representative panel study. Soc Psychiatry Psychiatr Epidemiol 2021; 56(5): 879–82. 33590312 10.1007/s00127-021-02032-w PMC8068655

[3] Auerbach RP, Mortier P, Bruffaerts R, Alonso J, Benjet C, Cuijpers P, WHO World Mental Health Surveys International College Student Project: prevalence and distribution of mental disorders. J Abnorm Psychol 2018; 127(7): 623. 30211576 10.1037/abn0000362 PMC6193834

[4] Gyimah S. Universities Must Ensure Their Mental Health Services Are Fit For Purpose. Department for Education, 2018 (https://educationhub.blog.gov.uk/2018/09/16/minister-gyimah-universities-must-ensure-their-mental-health-services-are-fit-for-purpose/).

[5] Fernandez A, Howse E, Rubio-Valera M, Thorncraft K, Noone J, Luu X, Setting-based interventions to promote mental health at the university: a systematic review. Int J Public Health 2016; 61(7): 797–807. 27364779 10.1007/s00038-016-0846-4

[6] Worsley J, Pennington A, Corcoran R. What Interventions Improve College and University Students’ Mental Health and Wellbeing? A Review of Review-Level Evidence. What Works Centre for Wellbeing, 2020 (https://whatworkswellbeing.org/wp-content/uploads/2020/03/Student-mental-health-full-review.pdf).

[7] Priestley M, Broglia E, Hughes G, Spanner L. Student perspectives on improving mental health support services at university. Couns Psychother Res 2022; 22(1): 1–10.

[8] Universities UK. #StepChange Mental Health in Higher Education. Universities UK, 2017 (https://www.universitiesuk.ac.uk/sites/default/files/field/downloads/2021-07/uuk-stepchange-mhu.pdf#page=12).

[9] Hughes GJ, Spanner L. The University Mental Health Charter. Student Minds, 2019 (https://universitymentalhealthcharter.org.uk/themes/).

[10] Hernández-Torrano D, Ibrayeva L, Sparks J, Lim N, Clementi A, Almukhambetova A, Mental health and well-being of university students: a bibliometric mapping of the literature. Front Psychol 2020; 11: 1226. 10.3389/fpsyg.2020.01226 PMC7296142 32581976

[11] Chalmers I, Bracken MB, Djulbegovic B, Garattini S, Grant J, Gülmezoglu AM, How to increase value and reduce waste when research priorities are set. Lancet 2014; 383(9912): 156–65. 24411644 10.1016/S0140-6736(13)62229-1

[12] Braun V, Clarke V. Reflecting on reflexive thematic analysis. Qual Res Sport Exerc Health 2019; 11(4): 589–97.

[13] Bengtsson M. How to plan and perform a qualitative study using content analysis. Nurs Plus Open 2016; 2: 8–14.

[14] Downe-Wamboldt B. Content analysis: method, applications, and issues. Health Care Women Int 1992; 13(3): 313–21. 1399871 10.1080/07399339209516006

[15] Burnard P. Interpreting text: an alternative to some current forms of textual analysis in qualitative research. Soc Sci Health 1995; 1(4): 236–45.

[16] Graneheim UH, Lundman B. Qualitative content analysis in nursing research: concepts, procedures and measures to achieve trustworthiness. Nurse Educ Today 2004; 24(2): 105–12. 14769454 10.1016/j.nedt.2003.10.001

[17] Krippendorff K. Content Analysis: An Introduction to its Methodology. Sage Publications, 2018.

[18] Broglia E, Ryan G, Williams C, Fudge M, Knowles L, Turner A, Profiling student mental health and counselling effectiveness: lessons from four UK services using complete data and different outcome measures. Br J Guid Counc [Epub ahead of print] 4 Feb 2021. Available from: 10.1080/03069885.2020.1860191.

[19] Barkham M, Broglia E, Dufour G, Fudge M, Knowles L, Percy A, Towards an evidence-base for student wellbeing and mental health: definitions, developmental transitions and data sets. Couns Psychother Res 2019; 4: 351–7.

[20] Bowyer K. A model of student workload. J High Educ Policy Manage 2012; 34(3): 239–58.

[21] Dyrbye LN, Thomas MR, Harper W, Massie Jr FS, Power DV, Eacker A, The learning environment and medical student burnout: a multicentre study. Med Educ 2009; 43(3): 274–82. 19250355 10.1111/j.1365-2923.2008.03282.x

[22] Slavin SJ, Schindler DL, Chibnall JT. Medical student mental health 3.0: improving student wellness through curricular changes. Acad Med 2014; 89(4): 573. 24556765 10.1097/ACM.0000000000000166 PMC4885556

[23] Jorm AF, Korten AE, Jacomb PA. “Mental health literacy”: a survey of the public's ability to recognise mental disorders and their beliefs about the effectiveness of treatment. Med J Aust 1997; 166: 182–6. 9066546 10.5694/j.1326-5377.1997.tb140071.x

[24] Jorm AF. Mental health literacy: empowering the community to take action for better mental health. Am Psychol 2012; 67(3): 231. 22040221 10.1037/a0025957

[25] Byrom NC. An evaluation of a peer support intervention for student mental health. J Ment Health 2018; 27(3): 240–6. 29451411 10.1080/09638237.2018.1437605

[26] Lo K, Gupta T, Keating JL. Interventions to promote mental health literacy in university students and their clinical educators. a systematic review of randomised control trials. Health Prof Educ 2018; 4(3): 161–75.

[27] Hughes GJ, Byrom NC. Managing student mental health: the challenges faced by academics on professional healthcare courses. J Adv Nurs 2019; 75(7): 1539–48. 30835889 10.1111/jan.13989

[28] Redpath J, Kearney P, Nicholl P, Mulvenna M, Wallace J, Martin S. A qualitative study of the lived experiences of disabled post-transition students in higher education institutions in Northern Ireland. Stud High Educ 2013; 38(9): 1334–50.

[29] Gulliver A, Farrer L, Bennett K, Griffiths KM. University staff mental health literacy, stigma and their experience of students with mental health problems. J Furt High Educ 2019; 43(3): 434–42.

[30] Andrews B, Wilding JM. The relation of depression and anxiety to life-stress and achievement in students. Br J Psychol 2004; 95(Pt 4): 509–21. 15527535 10.1348/0007126042369802

[31] Richardson T, Elliott P, Roberts R. Relationship between loneliness and mental health in students. J Public Ment Health 2017; 16(2): 48–54.

[32] Tembo C, Burns S, Kalembo F. The association between levels of alcohol consumption and mental health problems and academic performance among young university students. PLoS One 2017; 12(6): e0178142. 28658300 10.1371/journal.pone.0178142 PMC5489147

[33] Walters KS, Bulmer SM, Troiano PF, Obiaka U, Bonhomme R. Substance use, anxiety, and depressive symptoms among college students. J Child Adolesc Subst Abuse 2018; 27(2): 103–11.

[34] McIntyre JC, Worsley J, Corcoran R, Harrison Woods P, Bentall RP. Academic and non-academic predictors of student psychological distress: the role of social identity and loneliness. J Ment Health 2018; 27(3): 230–9. 29436883 10.1080/09638237.2018.1437608

[35] Peach H, Gaultney JF, Gray DD. Sleep hygiene and sleep quality as predictors of positive and negative dimensions of mental health in college students. Cogent Psychol 2016; 3(1): 1168768.

[36] Di Benedetto M, Towt CJ, Jackson ML. A cluster analysis of sleep quality, self-care behaviors, and mental health risk in Australian university students. Behav Sleep Med 2020; 18(3): 309–20. 30821507 10.1080/15402002.2019.1580194

[37] Karatekin C. Adverse childhood experiences (ACEs), stress and mental health in college students. Stress Health 2018; 34(1): 36–45. 28509376 10.1002/smi.2761

[38] Worsley JD, Harrison P, Corcoran R. Accommodation environments and student mental health in the UK: the role of relational spaces. J Ment Health [Epub ahead of print] 19 May 2021. Available from: 10.1080/09638237.2021.1922648. 34008464

[39] Worsley JD, Harrison P, Corcoran R. The role of accommodation environments in student mental health and wellbeing. BMC Public Health 2021; 21(1): 573. 33757482 10.1186/s12889-021-10602-5 PMC7986561

[40] Dogra S, MacIntosh L, O'Neill C, D'Silva C, Shearer H, Smith K, The association of physical activity with depression and stress among post-secondary school students: a systematic review. Ment Health Phys Act 2018; 14: 146–56.

[41] Hawkley LC, Cacioppo JT. Loneliness matters: a theoretical and empirical review of consequences and mechanisms. Ann Behav Med 2010; 40(2): 218–27. 20652462 10.1007/s12160-010-9210-8 PMC3874845

[42] Office for National Statistics (ONS). Loneliness - What Characteristics and Circumstances Are Associated with Feeling Lonely? ONS, 2018 (https://www.ons.gov.uk/peoplepopulationandcommunity/wellbeing/articles/lonelinesswhatcharacteristicsandcircumstancesareassociatedwithfeelinglonely/2018-04-10#:~:text=People%20in%20poor%20health%20or,reported%20feeling%20lonely%20more%20often.).

[43] Matthews T, Odgers CL, Danese A, Fisher H L, Newbury J B, Caspi A, Loneliness and neighborhood characteristics: a multi-informant, nationally representative study of young adults. Psychol Sci 2019; 30(5): 765–75. 30955415 10.1177/0956797619836102 PMC6512157

[44] Vasileiou K, Barnett J, Barreto M, Vines J, Atkinson M, Long K, Coping with loneliness at university: a qualitative interview study with students in the UK. Ment Health Prev 2019; 13: 21–30.

[45] Blackall M, Mistlin A. ‘Broken and Defeated’: UK University Students on the Impact of Covid Rules. The Guardian, 2021 (https://www.theguardian.com/education/2021/jan/11/broken-and-defeated-uk-university-students-on-the-impact-of-covid-rules).

[46] Montacute R, Culliane C. *Learning in Lockdown: Research Brief.* The Sutton Trust, 2021 (https://www.suttontrust.com/our-research/learning-in-lockdown/).

[47] Mountford-Zimdars A, Sanders J, Jones S, Sabri D, Moore J. Causes of Differences in Student Outcomes. Higher Education Funding Council for England, 2015 (https://www.researchgate.net/publication/324889905_Causes_of_differences_in_student_outcomes).

[48] Alharbi ES, Smith AP. Review of the literature on stress and wellbeing of international students in English-speaking countries. Int Educ Stud 2018; 11(6): 22–44.

[49] Lewis G, McCloud T, Callender C. Higher Education and Mental Health: Analyses of the LSYPE Cohorts. Department for Education, 2021 (https://www.gov.uk/government/publications/higher-education-and-mental-health-analyses-of-the-lsype-cohorts).

[50] Bewick B, Koutsopoulou G, Miles J, Slaa E, Barkham M. Changes in undergraduate students’ psychological well-being as they progress through university. Stud High Educ 2010; 35(6): 633–45.

[51] Macaskill A. The mental health of university students in the United Kingdom. Br J Guid Couns 2013; 41(4): 426–41.

[52] Eisenberg D, Hunt J, Speer N. Mental health in American colleges and universities: variation across student subgroups and across campuses. J Nerv Ment Dis 2013; 201(1): 60–7. 23274298 10.1097/NMD.0b013e31827ab077

[53] Eisenberg D, Golberstein E, Hunt JB. Mental health and academic success in college. BE J Econ Anal Policy 2009; 9(1): 1–40.

[54] Allan JF, McKenna J, Dominey S. Degrees of resilience: profiling psychological resilience and prospective academic achievement in university inductees. Br J Guid Couns 2014; 42(1): 9–25.

